# Histaminergic System and Inflammation-Related Genes in Normal Large Intestine and *Adenocarcinoma* Tissues: Transcriptional Profiles and Relations

**DOI:** 10.3390/ijms24054913

**Published:** 2023-03-03

**Authors:** Grażyna Janikowska, Tomasz Janikowski, Marta Plato, Urszula Mazurek, Joanna Orchel, Mieszko Opiłka, Zbigniew Lorenc

**Affiliations:** 1 Department of Analytical Chemistry, Faculty of Pharmaceutical Sciences in Sosnowiec, Medical University of Silesia, Jagiellońska 4 Street, 41-200 Sosnowiec, Poland; 2 Silesian College of Medicine in Katowice, Mickiewicza 29 Street, 40-085 Katowice, Poland; 3 Department of Molecular Biology, Faculty of Pharmaceutical Sciences in Sosnowiec, Medical University of Silesia, Jedności 8 Street, 41-206 Sosnowiec, Poland; 4 The Karol Godula Upper Silesian Academy of Entrepreneurship in Chorzów, Racławicka 23 Street, 41-506 Chorzów, Poland; 5 Katalyst Laboratories, London W1D 3QL, UK; 6 Clinical Department of General, Colorectal and Multiple Organ Trauma Surgery, Faculty of Health Sciences, Medical University of Silesia, Medyków 1 Square, 41-200 Sosnowiec, Poland

**Keywords:** *adenocarcinoma*, colorectal cancer, histaminergic system, inflammation, intestine, microarray, transcriptional analysis

## Abstract

Transcriptional analyses such as microarray data have contributed to the progress in the diagnostics and therapy of colorectal cancer (CRC). The need for such research is still present because of the disease being common in both men and women with a high second position in cancer rankings. Little is known about the relations between the histaminergic system and inflammation in the large intestine and CRC. Therefore, the aim of this study was to evaluate the expression of genes related to the histaminergic system and inflammation in the CRC tissues at three cancer development designs: all tested CRC samples, low (LCS) and high (HCS) clinical stage, and four clinical stages (CSI–CSIV), to the control. The research was carried out at the transcriptomic level, analysing hundreds of mRNAs from microarrays, as well as carrying out RT-PCR analysis of histaminergic receptors. The following histaminergic mRNAs: *GNA15*, *MAOA*, *WASF2A*, and inflammation-related: *AEBP1*, *CXCL1*, *CXCL2*, *CXCL3*, *CXCL8*, *SPHK1*, *TNFAIP6*, were distinguished. Among all analysed transcripts, *AEBP1* can be considered the most promising diagnostic marker in the early stage of CRC. The results showed 59 correlations between differentiating genes of the histaminergic system and inflammation in the control, control and CRC, and CRC. The tests confirmed the presence of all histamine receptor transcripts in both the control and colorectal *adenocarcinoma*. Significant differences in expression were stated for *HRH2* and *HRH3* in the advanced stages of CRC *adenocarcinoma*. The relations between the histaminergic system and inflammation-linked genes in both the control and the CRC have been observed.

## 1. Introduction

Despite the fact that colorectal cancer (CRC) is now a chronic disease (better treatment and survival), it is still the third cause of death in the world among all cancers [1,2]. CRC came second after lung cancer as the leading cause of cancer deaths in 2020 and its prevalence is not decreasing each year [2]. It is known that various factors contribute to the formation and development of CRC, including a diet low in fibre and calcium ions, alcohol abuse, obesity, and lack of physical activity [3,4,5]. Additionally, many mutations, which have been detected in genes controlling major signalling pathways, contribute to oncogenesis. These genes have been classified as oncogenes or tumour suppressors, correlating with changes detected in gene expression [5,6], including TP53, APC, BRAF, CTNNB, KRAS, PI3CA, and TGF-β mutations, and dysregulation of epithelial to mesenchymal transition (EMT) genes, activation of WNT signalling, and MYC amplification [6]. This knowledge contributed to better diagnostics and differentiation of CRC stages. Allowing the use of several gene tests enables a better understanding of this disease and increases patient survival [4,6]. However, there is still much to uncover that may increase the progress in both treatment and patient survival, especially diagnosis at an early stage.

Inflammation is a well-known risk factor for colorectal cancer [4]. Histamine and histaminergic response are connected with inflammation in the intestines and are associated with this cancer [6,7,8]. Both processes—inflammation and histaminergic response—appear as recurring symptoms of Crohn’s disease, which may affect the development and progression of CRC [7]. The influence of histamine and its receptors on the induction of the immune response and inflammation has been well documented in both gene and protein levels [8,9,10,11]. Although the presence of histamine in the intestine and its influence on the growth of cancer cells has been confirmed, its role in CRC has not been fully elucidated [8,9,12,13]. An important role of histamine in the development of CRC has been noticed in vascular neogenesis and imperfect homeostasis of histamine receptors [9].

The histamine *HRH1*, *HRH2*, *HRH3*, and *HRH4* receptor genes encode G protein-coupled receptors (GPCRs), which are also called H1R, H2R, H3R, and H4R, and are stimulated by histamine (biogenic amine) [14]. The receptors are coupled to different G protein subunits (alpha, beta, or gamma) that are involved as modulators or transducers in various signalling pathways. The alpha subunit of the guanine nucleotide binding protein is encoded by the *GNA* gene. H1R works through Gαq/11 protein and activates phospholipase C, which raises intracellular Ca^2+^ levels resulting among others, in smooth muscle contraction. H2R is a Gαs protein-coupled receptor that can induce mucus production and gastric acid secretion. H3R is coupled to Gαi/o protein, whereas H4R is also a Gαi/o protein-coupled receptor and shares 37% homology with H3R. All of them are an important part of the histaminergic system and can activate various signalling pathways [15,16,17,18,19]. HRH1 receptor signaling begins with the activation of phospholipase C (PLC), which activates protein kinase C (PKC) through the production of 1,2-diacylglycerol (DAG) and inositol-1,4,5-trisphosphate (IP3). Then, catalysing the phosphorylation of Ser/Thr mediators releases intracellular calcium. This results in an increase in PKC and the accumulation of cAMP (cyclic adenosine-3′, 5′-monophosphate) in the cell. The same is for H2R (an increase in calcium levels leads to an increase in PKC). However, in the case of H3R and H4R, a decrease in cAMP, an increase in calcium, and an increase in MAPK (mitogen-activated protein kinase) have been observed [18]. All of these mentioned proteins are encoded by their respective genes, the expression of which may vary with these changes. The pathophysiological role of histamine receptors and their signalling pathways (*PLC*/*IP_3_*/*DAG*/*PKC*, *ADCY*, and *MAPK*) is stimulated by them at the level of genes, and proteins, and plays an important role in the progression of cancer [19].

The genes encoding the mentioned receptors are present in most human tissues and also can be specific for a variety of human cells, for example, *HRH2* is mainly expressed in gastric cells, *HRH3* in cells of the central nervous system, and in the immune system of mast cells, T lymphocytes, and NK cells. *HRH4* is mainly present in hematopoietic cells, mast cells, eosinophils, dendritic cells, and regulatory T lymphocytes [17,18]. Histamine receptors regulate gastrointestinal motility and intestinal secretion [13,18]. *HRH1*–*HRH4* receptors have been found in neoplastic tissue and in the normal colon [20,21,22]. Decreases in *HRH1* and *HRH4* expression induced by histamine alone may be an indicator of local regulation disorders in colorectal tumours [20]. Both H1R and H2R are correlated with tumour growth, and blocking H1R and promoting H2R may reduce the risk of developing CRC in the population of patients at risk [10]. One study documented that the H3 receptor was specifically involved in the regulation of breast cancer growth and progression. H4R activation inhibited its proliferation, which was associated with a cell cycle arrest in the G1 phase and consequently, induction of apoptosis [23].

Histaminergic dysfunction can be caused by various factors at the gene or protein level, e.g., the concentration of histidine from which histamine is produced or diamine oxidase that can metabolise it. Moreover, diamine oxidase may influence the number of mast cells in which histamine is stored in an inactive form. Mast cells can build up in the gut lining, which can result in organ malfunction and/or carcinogenesis. It has also been proven that the key factors involved in the development of CRC are proangiogenic molecules such as histamine, vascular endothelial growth factor (VEGF), tumour necrosis factor α (TNFA), or interleukin (IL) 6 and 8 (CXCL8) [5,7,10,20,22,24,25].

Changes in the expression of genes of the histaminergic system (histamine receptors and associated cascade genes) and those related to inflammation have a significant impact on various signalling pathways and their dysregulation. Although the presence of histamine in the intestine has been confirmed along with its effect on histamine receptors, there are no studies describing the relationships between the histaminergic and inflammation-associated genes in the normal large intestine and CRC. Therefore, the aim of this study was to investigate the histaminergic and inflammation-related gene expression profiles at a transcriptomic level of 133, and 627 mRNAs, respectively, and their interrelationships in the normal large intestine, and CRC *adenocarcinoma*.

## 2. Results

### 2.1. Microarray Data Analysis

Microarrays are a technique that provides an opportunity to look at the expression of a large number of genes. When analysing the distribution of results, it is possible to see and compare their profiles.

Two lists of 133 histaminergic system (Table S1) and 627 inflammatory (Table S2) probes were used to perform two transcriptomes analysis in CRC (N = 18) and normal colon (N = 18) tissue samples. Normalised values (log transformation) show normality in the distribution of data (*p* ≤ 0.05). Descriptive statistics are presented as a box–whisker plots in Figure S1 which shows the distribution of normalised fluorescence intensity values (log_2_) as a median and 25th and 75th percentile for all probes from the used lists (Tables S1 and S2) with respect to all CRC samples (Figure S1A,B, respectively), two clinical stages, namely LCS (low clinical stage) and HCS (high clinical stage) (Figure S1C,D, respectively), and four clinical stages CSI–CSIV (Figure S1E,F, respectively), and the controls. The descriptive statistics show the presence of important, typical for the microarray analysis results. The location of large and small values and the presence of differences in each group of both transcriptomes are presented. For the mRNAs of the histaminergic system and inflammation in designs such as CRC, LCS, HCS, and CSI–CSIV, as well as the control, the profile plots are presented in Figure S2A–F, respectively. Additionally, the Supplementary Materials contain self-organizing maps in Euclidean distance metric conditions for all results of three designs of the histaminergic system and inflammation, which are presented in Figure S3A–F, respectively.

#### 2.1.1. Analysis of Differentially Expressed Genes

Differences in the distribution of significant probes (large and small values) are visible not only in Figure S1 but also in the number of significant mRNAs. The results of the moderated *t*-test (CRC) and ANOVA (LCS–HCS and CSI–CSIV) show the exact number of significant transcripts in each probability range. Regardless of the transcriptome, as the *p*-value increases the number of differentially expressed, mRNAs decrease, and additionally the Benjamini–Hochberg correction narrows them. The number of significant mRNAs for the 133 histaminergic system and 627 inflammation probes for different designs and *p*-values is presented in Table 1.

**Figure 1:**
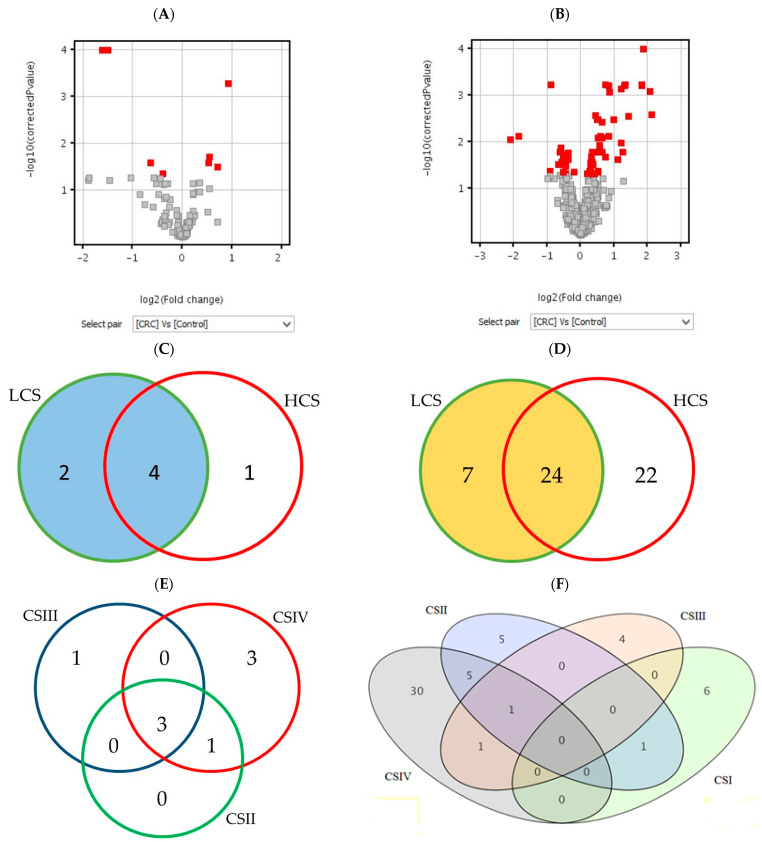
The number of differentially expressed mRNAs in the histaminergic system (**A**,**C**,**E**) and inflammation (**B**,**D**,**F**) in comparison of the CRC with control showed as volcano plots (**A**,**B**) and Venn diagrams (**C**–**F**). Legend: red gary squares (**A**,**B**)—significant results of the *t*-test; red squares in volcano plot—differentially expressed *t*-test significant results; circles or ellipses—individual stages of clinical advancement of CRC and the number of significant results; LCS—low clinical stage of colorectal cancer; HCS—high clinical stage of colorectal cancer; CSI–CSIV—clinical stages of colorectal cancer; the colours do not matter.

Figure 1 shows the number of statistically significant mRNAs in comparison to the control (BH corrected *p*-values), which were subjected to the moderated *t*-test (A and B) and Tukey HSD post hoc test (C–F). The moderated *t*-test results of the differentially expressed probes are presented as red squares in volcano plots. The Venn diagrams show a distribution of significant mRNAs in particular clinical stages of CRC. The highest number of differentially expressed mRNAs is in CSIV (the lowest cell differentiation and the presence of metastasis to other sites). The diagram for the histaminergic system has only three comparisons because of the lack of significant mRNA probes between CSI and the control (Figure 1E). In turn, when looking at the whole cancer formation process, many inflammation-related probes gave significant fluorescence signals in all clinical stages (Figure 1F), but not all of them met the criteria (all probes of the gene present on the microarray). All statistically significant mRNA probes of the histaminergic and inflammation-related genes (presented as quantities in Table 1, *p* < 0.05, and Figure 1) are presented in Table S3A–F in response to each research design.

All distinguished in these analyses (Figure 1A,C,E) results show differentially expressed genes related to the histaminergic system in CRC with a comparison to the control; those that met the criteria and presence in BH-corrected analysis are the following: *GNA15* (encoding G protein subunit alpha 15), *MAOA* (encoding monoamine oxidase A) and *WASF2* (encoding Wiskott–Aldrich syndrome protein family member 2).

Differentially expressed genes related to the inflammation in CRC with a comparison to the control (Figure 1B,D,F), those that met the criteria and are present in the BH-corrected analysis are the following: *AEBP1* (encoding AE binding protein 1 a member of carboxypeptidase A protein family), *CXCL1*, *2*, *3*, and *8* (encoding chemokines CXC-motif), *SPHK1* (encoding sphingosine kinase 1, which catalyses the phosphorylation of sphingosine to form sphingosine-1-phosphate), and *TNFAIP6* (encoding tumour necrosis factor alpha-induced protein 6).

The fold change (*FC*) and regulation of the distinguished mRNAs are presented in Table 2, which shows both the histaminergic system and inflammation (both uncorrected and corrected values) in whole samples (CRC), two (LCS and HCS), and four clinical stages (CSI–CSIV).

Unlike the *HRH2* and *HRH3*, the *HRH1*, and *HRH4* had a weak *FC* value for the uncorrected *p*-value, and not all of their probes were significantly expressed. Among the four histamine receptors, *HRH2* and *HRH3* are the only statistically significant when taking into consideration results from the microarrays without BH correction. Due to their important role in the histaminergic system, we took them into account in further analysis. Strong significance and an important role in the histaminergic response have also the following transcripts: *GNA15*, *MAOA*, and *WASF2* (all their probes expressed). The last two have decreased expression in all cancer stages compared to the control, while *GNA15* has increased expression.

All presented inflammation-related mRNAs (in Table 2) met the requirements of the high *p*-value (BH) and an *FC* greater than 2 in all the results design, and at least one clinical stage of the CRC development. The C-X-C motif chemokines have the highest *FC* values (*CXCL 1*–*3* in CSI and *CXCL8* in CSIV). The *AEBP1* displays potential for a good diagnostic gene. It has a low *FC* value in CSI in relation to the control and increases in subsequent stages. High *FC* and *p*-values of all outstanding mRNAs linked to inflammation confirm the presence of this process in the CRC *adenocarcinoma* development.

#### 2.1.2. Analysis of Correlation

Correlations between the expression of individual distinguished genes (the correlation coefficient—r is significant when the value of *p* is less than or equal to 0.05) are presented in Table 3. The obtained results confirm the presence of positive and negative correlations in the control and the CRC. In addition, their presence after correlating the expression of the highlighted genes in the control and CRC tissue was checked. As shown in Table 3, the correlations in the control (Table 3A) and/or tumour tissue (Table 3C) are stronger (larger absolute correlation coefficient r) than between the expression of genes in the control and tumour tissues of the intestine (Table 3B). Stronger negative correlations were observed in the control but were positive ones in cancer.

The positive correlations were presented in the normal large intestine between *HRH3* and *GNA15* or *CXCL3*, as well as *HRH4* and *MAOA*, *CXCL2* vs. *CXCL8*, or *TNFAIP6*, and others. Negative correlations were observed between *HRH4* and *WASF2* or *AEBP1*, or *SPHK1* (Table 3A).

Positive correlations between the expression of genes of the control and CRC were shown in *HRH2* and *GNA15* (CRC), as well as *WASF2* in the control, *AEBP1* or *TNFAIP6* in CRC, and others. Negative correlations were presented between *HRH4* in the control and *GNA15* or *TNFAIP6* in CRC, *CXCL3* (control), and *AEBP1* in CRC (Table 3B). The lower the expression of *HRH4* in the control tissue, the higher *GNA15* or *TNFAIP6* in CRC. These results may show the important role of the expression of these genes in the process of carcinogenesis at the borders of the normal large intestine and CRC.

Correlation analysis also shows that the following histaminergic transcripts: *GNA15*, *HRH2*, *MAOA*, *WASF2*, and those related to inflammation: *AEBP1*, *CXCL1*, *2*, *3*, and *8*, *SPHK1*, and *TNFAIP6* play an important role in cancer tissue.

Among them, positive correlations were showed *HRH2* with *GNA15*, *GNA15* with *CXCL8* or *SPHK1* or *TNFAIP6*, and *MAOA* with *WASF2*, but negative *HRH2* with *WASF2*, and *GNA15* with *MAOA* (Table 3C).

The results presented in Table 3 show 59 correlations for 7 histaminergic and 7 inflammation-related genes, with the most correlations for the control (24 and including 8 specifics to this comparison only), and CRC (20 and including 7 specifics to this comparison only). Correlations between gene expression in the control tissue and CRC (15 including 6 specifics for this comparison) are at a similar weak level (r = ±0.50), which is visible in part B.

**Figure 2:**
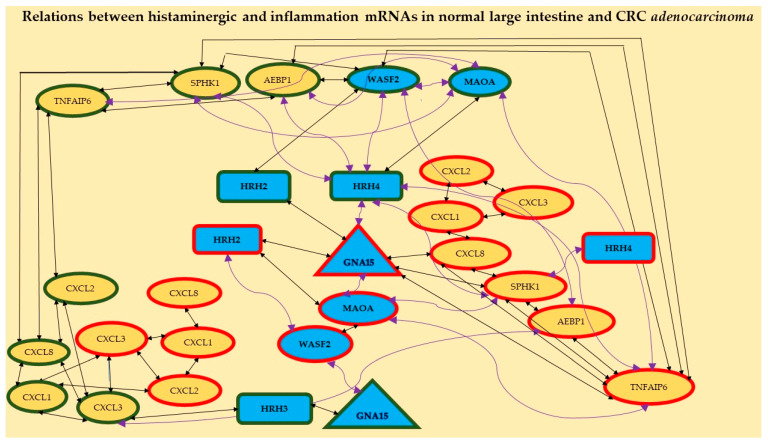
Relationships between distinguished mRNAs in both the histaminergic system and inflammation in the studied large intestine tissue (control) and CRC *adenocarcinoma*. Legend: rectangles—receptors, triangle—molecule *GNA15*, ellipses—different cascade molecules; green contour—control intestine tissue, red contour—CRC; blue background and bolded font—histaminergic system, yellow background—inflammation-related molecules; black straight lines—positive correlation; violet twisted lines—a negative correlation. The different symbol forms are presented for better visualisation of the results.

Moreover, different ways of *HRH2* and *HRH4* action in the studied CRC are shown in Figure 2. The first one probably acts through the histaminergic system and the second through inflammation; the higher the expression of *HRH2*, the lower the expression of *WASF2*; the lower the expression of *HRH4*, the higher the expression of *SPHK1*. Figure 2 confirms the relationships between the expressions of histaminergic and inflammation-linked genes in the control tissue (eight correlations) and CRC (seven correlations).

Additionally, it shows that receptors *HRH2*, *HRH3*, and *HRH4* play an important role in normal intestinal tissue but *HRH2* and *HRH4* are important in CRC *adenocarcinoma*. *HRH1* showed no correlation with any gene, regardless of tissue.

### 2.2. Expression Profile of Histaminergic Receptors

**Figure 3:**
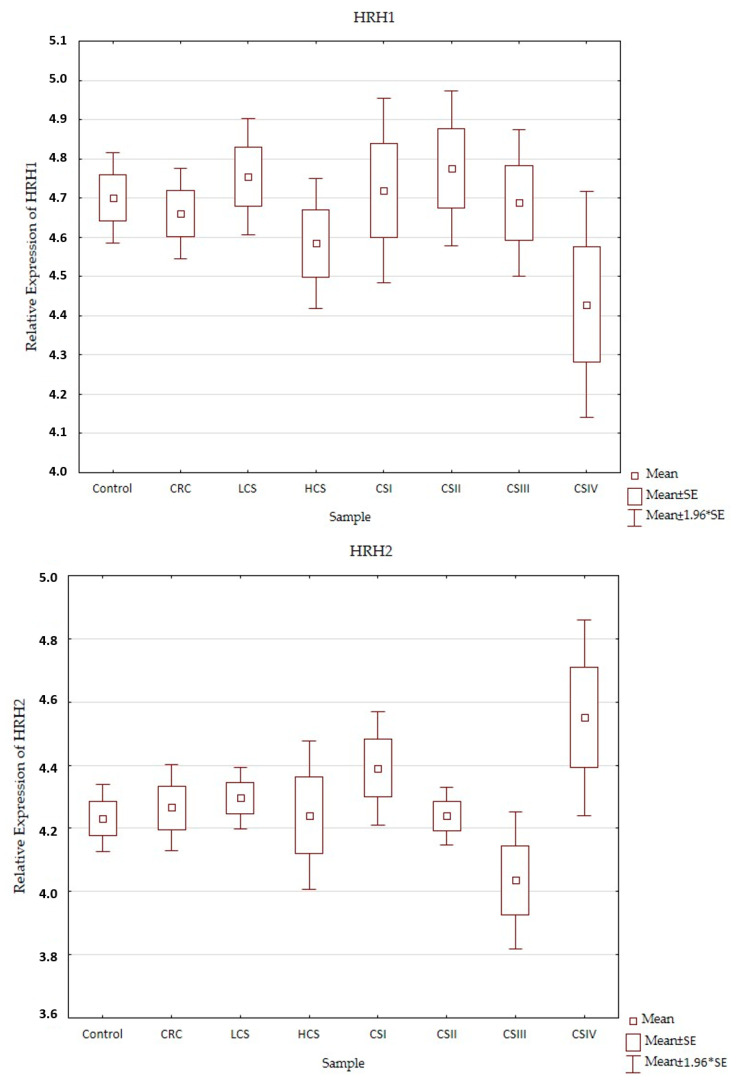

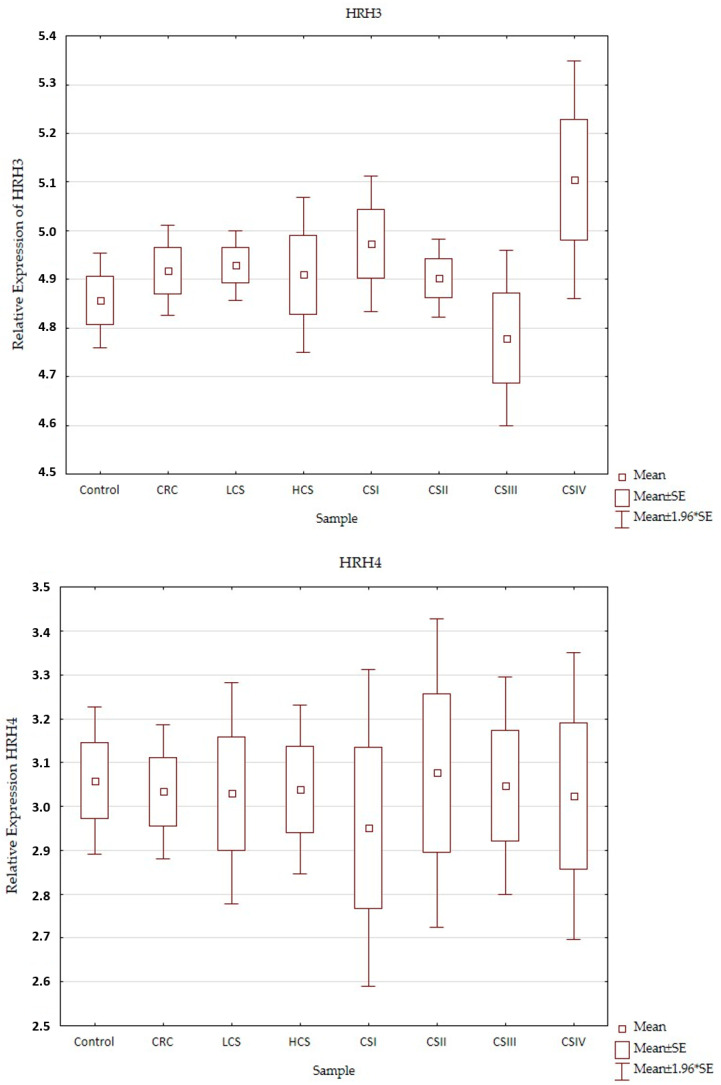
Profile of relative expression of histamine receptors in the normal large intestine (control) and colorectal cancer (CRC, LCS–HCS, CSI–CSIV). Legend: *HRH1*—A, *HRH2*—B, *HRH3*—C, *HRH4*—D; CRC—all CRC samples; LCS—low clinical stage and HCS—high clinical stage; CS –clinical stage; CSI—clinical stage I; CSII—clinical stage II; CSIII—clinical stage III; CSIV—clinical stage IV; SE—standard error; mean ±1.96 * SE—mean with confidence interval.

Expression profiles of the histamine receptors (*HRH1*–*HRH4*) in the control and CRC in all three designs (CRC, LCS–HCS, and CSI–CSIV) are presented in Figure 3. Comparisons of the tested samples (controls, CRC, LCS, HCS, CS I–IV—together) did not show statistically significant changes. A single comparison of CRC to the control or LCS and HCS to the control showed no significant changes for any of the receptors tested.

However, when comparing the individual stages of CRC (CSI–CSIV) and the control, only the *HRH4* expression profile did not show significant changes between all examined samples (control and histamine receptors in the CRC development). Significant differences were present in the expression profile of *HRH1* between CSII and CSIV (*p* = 0.042), and *HRH2* between CSIII and CSIV (*p* = 0.025), as well as *HRH3* between the control and CSIV (*p* = 0.03). All the presented samples had a low expression (even for the control). *HRH2* and *HRH3* expression increased in CRC development and had the highest significant expression for CSIV, but *HRH1* decreased. Despite the fact that the expression of histamine receptors in all the tested samples was weak, the *HRH4* had the lowest expression, regardless of sample kind at a similar level.

## 3. Discussion

The assessment of changes in gene expression in CRC adenocarcinoma at various designs of results allowed us to select from all differentiating genes those that can have high importance in particular stages of clinical advancement and may contribute to the progress of early cancer diagnosis. The presented analysis of CRC samples in comparison to the normal large intestine at the transcriptional level showed an increase in the number of mRNAs, which was changing the intensity of fluorescence (decrease or increase in relation to control) with the development of cancer. The largest amount of mRNA differentiating cancer from control regardless of the transcriptome was observed in the highest clinical advancement. Taking into consideration the cancer development process, the following histaminergic genes stood out: *GNA15*, *HRH1*-*HRH4*, *MAOA*, *WASF2*, along with inflammation-related genes: *AEBP1*, *CXCL1*, *CXCL2*, *CXCL3*, *CXCL8*, *SPHK1*, and *TNFAIP6*. In addition, 59 correlations between these genes in histopathologically normal colon and CRC *adenocarcinoma* were presented. Taking into consideration the number of genes involved in both processes and differentiation of the various stages of the disease showed the dominance of inflammation, which manifested by an increase in *FC* and *p*-values for distinguished chemokines in all the results designs. These chemokines are well-described in different cancers and are actively involved in the proliferation, stemness, and survival of neoplastic cells [22]. In our study, the correlations between all the distinguished chemokines: *CXCL1*, *CXCL2*, *CXCL3*, and *CXCL8* were presented for the first time (Figure 2). As CRC develops, the expression of the first three chemokines decreases, while *CXCL8* expression increases. It was recently discovered that *CXCL8* is a good blood marker in the progression of colorectal cancer [26,27]. Moreover, the participation of these chemokines in the development of CRC has been demonstrated in a mouse model [9] and human cells [10,11]. Our findings confirm these previously presented results.

Furthermore, an upward trend in CRC development was observed in the expression of the histaminergic system-related gene—*GNA15* and inflammation—*AEBP1*, *SPHK1*, and *TNFAIP6*. In contrast, the *MAOA* expression decreases with the development of cancer. Several studies confirmed the relationship between histaminergic and inflammation molecules during the development of neoplastic processes [10,28,29,30,31,32]. Contradictorily, the relationship between two histaminergic receptors H1R (promote) and H2R (suppress) in gut inflammation and colonic carcinogenesis was presented in the mouse model, while the ratio of *HRH2*/*HRH1* gene expression was significant in the human colorectal cancer-derived cell lines (HCT116, Caco2, DLD1, LS174T, and HT29) [10]. The role of *HRH4* in breast cancer cell proliferation [28] and expression of *GNA15*, and different chemokines in small intestinal neuroendocrine neoplasia [29], pancreatic ductal *adenocarcinoma* [30], and ovarian cancer [31] was also observed. However, for the first time, so many correlations between histaminergic and inflammation-related genes were shown in the normal large intestine and CRC in our study. The correlation analysis of data from microarray showed that *HRH2*, *HRH3*, and *HRH4* play a leading role in the control (histopathologically normal large intestine tissue), while *HRH2* and *HRH4* play a leading role in the CRC. On the other hand, the main role in breast cancer [28] and melanoma [32] was attributed to *HRH4*, which in the presented results, had the lowest expression in all stages. It was stated that blocking HRH4 by its antagonists may inhibit cancer cell proliferation [33] and its deficiency also reduced CRC development in experimental mice [34]. Decreased expression of this gene may be a good prognosis.

Positive correlation in colon control tissue *HRH2* and *GNA15*, which encodes a heterotrimeric G protein, Gα15, and belongs to the Gαq subfamily, confirms the fact of their direct cooperation [16,17]. The presence of this gene was observed in pancreatic ductal *adenocarcinoma* [30,35], ovarian cancer [31], and oesophagus squamous cell carcinoma [36]. Zanini et al. showed that *GNA15* expression in the gastrointestinal neuroendocrine neoplasia of the small intestine can promote cell proliferation and inhibit cellular apoptosis [29]. A similar effect can be expected in the presented study, in which *GNA15* in subsequent stages of colorectal *adenocarcinoma* had increased expression. In contrast, expression of *GNA15* in the pancreas was limited to transformed cells and occurred in the initial stages of pancreatic ductal *adenocarcinoma* progression [35]. Besides our research, the role of *GNA15* in CRC development is unknown. However, many relations between its expression and other genes related to the histaminergic system and/or inflammation indicate its important role in these processes. The presented findings show positive correlations between *HRH3* and *GNA15*, and *CXCL3* only in the control, which confirms their relation in these cells. The positive correlation between *HRH2* and *GNA15* in CRC indicates the same mechanism of action of this receptor in both colon tissues. The possible relation between histaminergic and inflammation-related genes also shows positive correlations between *GNA15* and *CXCL8* (also called *IL8*), *SPHK1*, and *TNFAIP6* in the CRC. But negative correlations of *GNA15* with *MAOA* and *MAOA* with *SPHK1*, and *TNFAIP6* confirm an important role of these molecules in CRC advancement. Moreover, a negative correlation between *HRH2* and *WASF2* in the CRC and a positive correlation in the control indicate this molecule is critical and variable depending on the microenvironment. It confirms also that *WASF2* is a main mediator of cancer progression via the histaminergic system with *HRH2* in the CRC. Specifically, the higher the *HRH2* expression, the lower the *WASF2* expression (increased expression of *WASF2* will decrease *HRH2*)*. WASF2* encodes Wiskott–Aldrich syndrome protein family verprolin-homologous protein 2 (other names *WAVE2*, *WASP*, *SCAR2*). Other findings showed that *WASF2* can participate in the cytoskeleton remodelling of the CRC microenvironment and liver metastasis [37,38]. Expression of *WASF2* was detected in colonic cancer cells, but not in normal colonic epithelial cells [39]. *WASF2* is overexpressed in lung *adenocarcinoma* [40], breast carcinoma [41], pancreatic cancer [42], hepatocellular carcinoma [43], ovarian cancer [44], and its high expression is associated with poor prognosis, treatment resistance, and metastasis. The presented results confirmed a greater expression of the *WASF2* in the control tissue than in CRC *adenocarcinoma* (Table 2), which may indicate a good prognosis for the patients.

The analysis also showed that the control *HRH4* may correlate positively with *MAOA* and negatively with *WASF2*, and inflammation-linked transcripts such as *AEBP1* and *SPHK1*. However, the *HRH4* (control) has a negative relation with CRC histaminergic *GNA15* and inflammation-related *SPHK1* and *TNFAIP6*, but CRC *HRH4* only has a negative relation with *SPHK1* CRC.

*MAOA* is diminished in the examined CRC. This gene encodes monoamine oxidase A enzyme which catalyses the oxidative deamination of the main biogenic amines: dopamine (precursor to norepinephrine), norepinephrine (anxiety-related molecule), and serotonin. *MAOA* was also downregulated in hepatocellular carcinoma patients [45]. Low expression was observed in normal secretory prostatic epithelium and in low-grade prostate cancer. Contrarily, high expression of *MAOA* in the normal basal prostatic epithelium and in high-grade primary prostate cancer was found [46]. Similar to our findings, continuously downregulated *MAOA* was also found in different colon tumours [47]. Contrary to the control intestine, a positive correlation between *MAOA* and *WASF2* was observed in the tested CRC that confirms the important role of these molecules in the cancerogenesis of the large intestine.

In contrast, the inflammation-related gene *AEBP1* encodes adipocyte enhancer-binding protein 1 from the carboxypeptidase A protein family, which can act as a transcriptional repressor and modulator of inflammation. Hence, its overexpression is associated with various cancer types [48]. Research suggests that the upregulation of *AEBP1* can contribute to tumour angiogenesis in primary CRC [49]. This is partially in line with the presented results due to the increased expression of *AEBP1* in the CSII–CSIV, but not in the first stage of the studied cancer, where decreased expression of this mRNA was observed. Such expression (low expression in the early stage of CRC) can indicate that the *AEBP1* could become a valuable diagnostic marker for CRC, but further research is needed.

Next, *SPHK1* encodes sphingosine kinase 1, which phosphorylates sphingosine to sphingosine-1-phosphate (S1P signalling) and was overexpressed in various types of cancers, as well as in CRC tissues and cell lines [50]. Increased expression of *SPHK1* is associated with lymph node and liver metastasis, and advancement in the TNM stage [51]. Similar to the presented results, the *SPHK1* showed upward trends in expression from CSI to CSIV. The connection of this inflammation-linked transcript with the histaminergic system genes manifested in a positive relation with *WASF2* in the control. Unchangeable, negative relations of the *HRH4* with *SPHK1* were observed in both the control and *adenocarcinoma* colon. Additionally, *SPHK1* (CRC) had a positive correlation with *GNA15* (CRC) and a negative correlation with *MAOA* in the CRC. This molecule correlates with *HRH4* but not with *HRH2* or *HRH3* in the control.

In turn, *TNFAIP6* which encodes tumour necrosis factor alpha-induced protein 6, is also known as *TNF* stimulated gene (TSG-6) and is an inflammation-associated molecule. Its role is mainly in mediating immunomodulatory activities in mesenchymal stem/stromal cells [52]. *TNFAIP6* has been found to be differentially expressed in CRC patients’ blood [53] and also upregulated in CRC tissue [54]. Our findings show enhanced expression of this transcript in the advanced stage (CSIII and CSIV) and its probable connection with histaminergic system genes (positive correlation with *GNA15* and negative with *MAOA*) in the tested CRC. The mentioned relations for *SPHK1* and *TNFAIP6* may suggest that the reduction in *MAOA* expression could affect the progress of inflammation in CRC.

Analysing the presented results, it can be speculated that the neoplastic process in the large intestine initiates the relation of *WASF2* and *HRH2* through indirect participation of *GNA15*, and this feedback can be with the participation of pro-inflammatory *IL8* (*CXCL8*), as well as *SPHK1* and *TNFAIP6*.

Hence, the histamine receptors were validated and included in the analysis but did not show statistically significant differences. The relative expression of the histaminergic receptors showed significant differences only for *HRH1*, *HRH2*, and *HRH3* in particular stages of CRC development, CSII vs. CSIV, CSIII vs. CSIV, and control vs. CSIV, respectively. Other investigation shows that *HRH1*, *2*, and *4* expressions were demonstrated in CRC and normal mucosa, with *HRH1* and *4* being decreased in CRC [21]. Similar to our findings, mRNA levels of *HRH4* were also reduced in both early-stage and advanced CRC samples [55]. Another study stated that *HRH4* expression in CRC tissue and normal colon mucosa was present, and *HRH1*, *HRH2*, and *HRH4* in human colon cancer cells: HT29, Caco-2, and HCT116 [56]. Expression of histamine receptors *HRH1*–*HRH4* was also presented in human pancreatic cancer cells [13,57] and breast, ovarian, and many others [28]. Many of these studies suggest that receptor response is specifically related to the cell or tissue type, but also to other factors such as agonist concentration and/or the coexistence of other molecules [21,28,32,57]. The reason for the changes in the expression of these molecules may have been due to the individuality of each study.

The studies confirmed the presence of all four histamine receptors in the tissue of the histopathologically unchanged intestine (control), as well as CRC. Furthermore, these studies for the first time highlight the correlations between the histaminergic and inflammation genes in both the control tissue and CRC *adenocarcinoma*.

## 4. Materials and Methods

The material for the investigation were samples of the large intestine collected from patients during surgical procedures at the Clinical Department of General, Colorectal, and Trauma Surgery of the Medical University of Silesia. The demographic and clinical characteristics of the participants taking part in the studies are presented in Table 4.

All the patients’ preoperative diagnostic tests were within normal limits. The haemoglobin of the patients was in the normal range (Table 4), and the patients did not require any blood transfusions before the operation. A total of 25% of patients with advanced cancer (CIII and CSIV) required transfusion after the surgery.

The samples for analysis were visually assessed by at least two surgeons and were obtained from the central part of the tumours. The intestine sections were classified as normal colon tissues (control) and as colorectal cancer (CRC). Samples for analysis were obtained from patients clinically in different stages of disease during the resection of the large intestine. Samples were collected from both men and women patients for the 97 CRC samples. The 19 control samples were collected from colon tissue outside the margin of the macroscopically and histopathologically assessed as a normal large intestine (control). Until the analysis was performed; the samples were stored in a freezer at −80 °C. All patients were from the industrial area of Poland named Upper Silesia.

The project of the study was approved by the Bioethics Commission of the Medical University of Silesia (KNW/0022/KB1/21/I/10) and conforms to the standard set by the Declaration of Helsinki (printed in the *British Medical Journal*, 1964, and next changes). Written informed consent for the use of their material to study was obtained from all participants.

### 4.1. Selection of Material for Analysis

**Figure 4:**
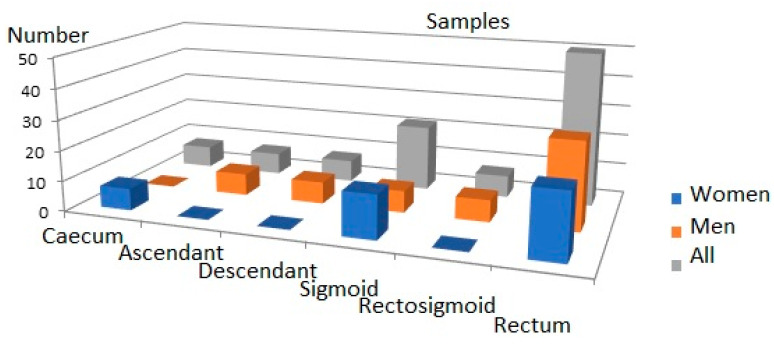
Distribution of tumours in large intestine.

The criteria for exclusion from the study group included patients: treated with histaminergic inhibitors and hormonal drugs in the last year before surgery, diagnosed with a form of cancer other than CRC, with unclear histological confirmation of CRC, re-operated on due to the CRC, as well as with genetic, metabolic, or systemic conditions (as obesity, hyperlipidaemia, hyperglycaemia), with any allergic symptoms, after previous radio- and/or chemotherapy and undergoing or after hormone replacement therapy completed within the 5 years prior to the operation. The criteria for inclusion were CRC *adenocarcinoma* in histopathological assessments of tumour material obtained in post-surgery of patients in all stages of the disease undergoing elective classical surgical procedures. The criteria for inclusion and exclusion were found in 36 studied cases. The tumours’ locations were varied (caecum, ascending colon, descending colon) in the large intestine and the distribution of the samples is presented in Figure 4.

Among the tested samples, there were 18 *adenocarcinomas* of the colon (CRC) and 18 controls. CRC samples were divided into two groups: LCS—low stage (N = 9) and HCS—high stage (N = 9).

Taking into consideration the 7th edition of the AJCC (American Joint Committee of Cancer)/UICC staging system of CRC with the next changes [58,59], the cancer tissues were divided into 4 groups: clinical stage I (CSI, N = 4), clinical stage II (CSII, N = 5), clinical stage III (CSIII, N = 5), and clinical stage IV (CSIV, N = 4), which is explained in Table 5. For practical interpretation and visualisation, see https://www.cancer.net/cancer-types/colorectal-cancer/stages.

### 4.2. Molecular Analysis of Samples

The molecular analysis was started by extracting total RNA from the collected fragments of the large intestine stored in RNAlater (Qiagen, Hilden, Germany) at a temperature of −80 °C. In further stages of the study, RNA was the array for the assessment of intestinal transcriptome, using expression microarrays HG-U133A (Affymetrix^®^, Santa Clara, CA, USA) and validation of selected transcripts.

#### 4.2.1. RNA Extraction and Purification

Total RNA from the 36 samples of the large intestine was isolated with TRIzol^®^ reagent (Invitrogen, Carlsbad, CA, USA) according to the instruction. Next, the extracted RNA was purified using columns of RNase Mini kit (Qiagen, Valencia, CA, USA) in accordance with the manufacturer’s instructions. A qualitative assessment of the obtained RNA extract was performed using 1% agarose gel electrophoresis, stained with ethidium bromide. Additionally, the degree of total RNA integrity was assessed based on the RNA Integrity Number (RIN) parameter—rRNA ratio (28 s/18 s). RNA concentration was assessed spectrophotometrically at a wavelength of 260 nm, using a Gene Quant II spectrometer (Pharmacia BioTech, Uppsala, Sweden).

#### 4.2.2. Microarray Hybridization

The isolated total RNA was used for the synthesis of marked cRNA (biotinylated complementary RNA), the synthesis of which was performed using the 3′ IVT Express Kit. The obtained cRNAs were hybridised with HG-U133A microarrays. At the next stage, the microarrays were washed and marked by immunofluorescence using the Fluidics Station 450 and the Hybridization Wash and Stain Kit. Next, the fluorescence intensity of the transcriptomes was read using the GeneChip Scanner 3000 7G and the Affymetrix^®^ GeneChip^®^ Command Console^®^ software. Experiment quality control was carried out in the subsequent stages of transcriptome assessment as reverse transcription products (cDNA), transcription (cRNA), and cRNA after fragmentation and immediately before the preparation of the hybridization cocktail. Microarray quality control tests were carried out using the above-mentioned software. Analysis of the 22,283 mRNAs was performed and the degradation index of RNA was assessed using a 3′/5′ ratio (signal intensity ratio of the 3′ probe set over the 5′ probe set) of the GAPDH and ACTB, for the following probes: AFFXBioB_at, AFFXBioC_at, AFFXBioDn_at, AFFXCreX_at, AFFXr2EcBioB_at, AFFXr2EcBioC_at, AFFXr2Ec-BioD_at, and AFFXr2P1cre_at.

#### 4.2.3. Relative Expression of Selected Transcripts

A precise assessment of diagnostic and prognostic values of the determined changes in mRNA concentration of HRH1, HRH2, HRH3, and HRH4 was carried out. The RT-PCR reaction was conducted using the SYBR Green Quantitect RT-PCR kit (Qiagen, Valencia, CA, USA) and the Opticon™ DNA Engine Sequence Detector (MJ Research Inc., Watertown, MA, USA). Starters sequence (5′ → 3′) for *HRH1* (GenBank Access. No. D28481.1 forward primer ATGCCGTACGGAGTGAGCGG, reverse primer GCTGGACAGAGCGGTAGCGA product length 243 bp), *HRH2* (GenBank Access. No. AY136744.1 forward primer ACCGCATCTTCAAGGTCGCC, reverse primer GTTGGCATAGCCCAGCCACA product length 233 bp), *HRH3* (GenBank Access. No. NM_007232.3 forward primer GGCCACTGCGTCCCTGACTA, reverse primer TGGTGGGCCACTCACTTCCA product length 194 bp), *HRH4* (GenBank Access. No. NM_021624.4 forward primer ACATCCCTCACACGCTGTTCG, reverse primer ACCCAAACGGCCACCATCAG product length 208 bp) *ACTB* (GenBank Access. No. NM_001101 forward primer TGACGTGGACATCCGCAAAG, reverse primer CTGGAAGGTGGACAGCGAGG product length 205 bp) were synthesised by Oligo IBB PAN (Warszawa, Poland). The RT-PCR reaction was conducted using the SYBR Green Quantitect RT-PCR kit (Qiagen, Valencia, CA, USA) and the Opticon™ DNA Engine Sequence Detector (MJ Research Inc., Watertown, MA, USA). The number of mRNA copies in 1 μg of the total RNA extract was determined based on the standard curve made for commercially available DNA specimens of the β-actin gene. For each test, negative control (without RNA array) and endogenous control with mRNA of β-actin were carried out. The specificity of the RT-PCR reaction was assessed based on the electrophoretic separation of amplimers at 6% polyacrylamide gel stained with silver salts. Tm value was determined based on the amplimer melting curve, for each RT-PCR product. Data were normalised using the 2−∆∆Ct method.

### 4.3. Statistical Analysis

Statistical analysis of the obtained data was performed using Statistica 13.0 (StatSoft sp. z o.o., Kraków, Polska), Excel 2007 (Microsoft Co., Redmond, WA, USA) and GeneSpring 13.0 (Agilent Technologies Inc., Santa Clara, CA, USA) software.

Fluorescence signals of 22,283 mRNAs for each HG-U133A chip were normalised using the RMA (Robust Multichip Average) method. The analyses were carried out on data obtained from control (N = 18) and CRC (N = 18) samples collected from patients during surgery. From the total mRNA, probe sets were selected 133 related to the histaminergic system and 627 to inflammation. The Shapiro–Wilk test was used to check the normality of the data distribution.

For each selected transcriptome, GeneSpring 13.0 software was used according to the manual. Similarities and differences between samples were checked by the use of descriptive statistics and one-way analysis of variance with the Tukey post hoc test. For analysis of these data, the Benjamini–Hochberg correction was used with a false discovery rate (FDR *p*-value < 0.05) to better control the procedure [60]. Additionally, the fold change (*FC*) parameter was greater than 2.0 (*FC* > 2.0) at least once in all the stages of CRC or less, when it was significant for analysis. The visualisation of high significance differences obtained in the moderated *t*-test (CRC design) and post hoc Tukey HSD test (LCS and HCS, and CSI–CSIV design) for differentiated transcripts was presented as volcano plots, as well as Venn diagrams. All differences were statistically significant if the *p*-value was less than 0.05 (*p* < 0.05). For all distinguishing genes, a Pearson correlation analysis was performed in Statistica 13.0 assessing the values of the correlation coefficients (r) as significant when the *p*-value was less than or equal to 0.05 (*p* ≤ 0.05).

## 5. Conclusions

Among the histaminergic system and the inflammation-related genes, the following genes were significantly expressed: *GNA15*, *HRH1*-*HRH4*, *MAOA*, *WASF2* and *AEBP1*, *CXCL1*, *CXCL2*, *CXCL3*, *CXCL8*, *SPHK1*, and *TNFAIP6* in the tested samples. *AEBP1* could be considered a candidate for an early biomarker of CRC *adenocarcinoma.*

The presented results showed that the profiles of statistically significant mRNAs related to the histaminergic system and inflammation involved in these processes change with the development of CRC.

Despite the observed involvement of both investigated transcriptomes in the development of CRC, it can be concluded that inflammation plays a leading role in it (larger responses and differences, as well as more correlations).

Correlative analysis showed significant relations between the expression of the distinguished histaminergic system and inflammation-linked genes.

Besides the *HRH4*, the importance of histaminergic receptors increases with the development of CRC.

## Figures and Tables

**Table 1:** The number of significant mRNAs related to the histaminergic system and inflammation in relation to uncorrected *p*-value and Benjamini–Hochberg correction in response to research design.

Probes	Design	*p*-Value	*p* < 0.05	*p* < 0.02	*p* < 0.01	*p* < 0.005
Histaminergic system	CRC	uncorr	33	25	18	13
		BH	10	4	4	4
	LCS–HCS	uncorr	31	20	14	9
		BH	7	6	6	6
	CSI–CSIV	uncorr	24	18	13	10
		BH	10	9	7	5
Inflammation	CRC	uncorr	154	108	78	62
		BH	62	34	24	18
	LCS–HCS	uncorr	156	99	80	59
		BH	53	26	22	14
	CSI–CSIV	uncorr	181	114	84	66
		BH	71	39	32	24

Legend: *p*—probability value; CRC—colorectal cancer (all the test samples in one); uncorr—uncorrected *p*-value; BH—Benjamini–Hochberg correction of false discovery results; LCS—low clinical stage, HCS—high clinical stage (a division of the test samples into two cancer stages); CS—clinical stage (a division of the test samples into four cancer stages).

**Table 2:** Fold change of distinguished mRNAs related to histaminergic system and inflammation in CRC development.

Probes	Gene Symbol	Probe Set ID	Clinical Stage, Fold Change, Regulation												
			*FC* CRC	*p*	BH	*FC* LCS	*FC* HCS	*p*	BH	*FC* CSI	*FC* CSII	*FC* CSIII	*FC* CSIV	*p*	BH
Histaminergic system	*GNA15*	205349_at	1.4 ↑	1 × 10^−3^	3 × 10^−2^	1.3 ↑	1.6 ↑	2 × 10^−2^	3 × 10^−2^	1.2 ↑	1.3 ↑	1.4 ↑	2.0 ↑	1 × 10^−3^	2 × 10^−2^
	*HRH2*	220805_at								1.1 ↑	1.0 ↑	−1.1 ↓	1.2 ↑	3 × 10^−2^	
	*HRH3*	220447_at								1.1 ↑	1.1 ↑	−1.0 ↓	1.4 ↑	3 × 10^−2^	
	*MAOA*	204388_s_at	−2.8 ↓	8 × 10^−9^	6 × 10^−7^	−3.2 ↓	−2.4 ↓	3 × 10^−8^	4 × 10^−6^	−1.6 ↓	−3.1 ↓	−2.7 ↓	−4.1 ↓	5 × 10^−9^	3 × 10^−7^
	*MAOA*	204389_at	−2.8 ↓	3 × 10^−7^	1 × 10^−5^	−3.3 ↓	−2.8 ↓	6 × 10^−8^	4 × 10^−6^	−1.6 ↓	−3.9 ↓	−2.5 ↓	−4.8 ↓	8 × 10^−10^	1 × 10^−8^
	*MAOA*	212741_at	−3.0 ↓	9 × 10^−9^	6 × 10^−7^	−3.2 ↓	−2.5 ↓	1 × 10^−6^	5 × 10^−5^	−1.6 ↓	−3.4 ↓	−2.5 ↓	−4.6 ↓	1 × 10^−7^	6 × 10^−5^
	*WASF2*	221725_at	−1.5 ↓	5 × 10^−3^		−1.4 ↓	−1.6 ↓	2 × 10^−2^		−1.7 ↓	−4.2 ↓	−1.2 ↓	−2.4 ↓	5 × 10^−3^	9 × 10^−3^
Inflammation	*AEBP1*	201792_at	2.4 ↑	9 × 10^−4^	2 × 10^−2^	1.5 ↑	3.4 ↑	3 × 10^−4^	9 × 10^−3^	−1.1 ↓	2.1 ↑	3.5 ↑	3.2 ↑	8 × 10^−5^	1 × 10^−2^
	*CXCL1*	204470_at	3.5 ↑	8 × 10^−6^	6 × 10^−4^	4.8 ↑	2.8 ↑	1 × 10^−5^	1 × 10^−3^	6.7 ↑	3.9 ↑	2.6 ↑	3.1 ↑	2 × 10^−5^	1 × 10^−3^
	*CXCL2*	209774_x_at	4.2 ↑	1 × 10^−5^	8 × 10^−4^	5.2 ↑	3.5 ↑	4 × 10^−5^	2 × 10^−3^	9.2 ↑	3.7 ↑	4.4 ↑	2.4 ↑	3 × 10^−5^	1 × 10^−3^
	*CXCL3*	207850_at	3.5 ↑	4 × 10^−6^	6 × 10^−4^	5.0 ↑	2.7 ↑	4 × 10^−6^	6 × 10^−4^	8.7 ↑	3.6 ↑	2.6 ↑	2.8 ↑	2 × 10^−6^	2 × 10^−4^
	*CXCL8*	202859_x_at	4.4 ↑	5 × 10^−5^	3 × 10^−3^	4.2 ↑	4.6 ↑	2 × 10^−4^	8 × 10^−3^	3.9 ↑	4.3 ↑	3.3 ↑	7.3 ↑	4 × 10^−4^	7 × 10^−3^
	*SPHK1*	219257_s_at	1.7 ↑	5 × 10^−6^	6 × 10^−4^	1.3 ↑	2.1 ↑	4 × 10^−8^	2 × 10^−5^	1.2 ↑	1.3 ↑	1.8 ↑	2.6 ↑	6 × 10^−9^	4 × 10^−6^
	*TNFAIP6*	206025_s_at	2.3 ↑	4 × 10^−4^	1 × 10^−2^	1.5 ↑	3.3 ↑	8 × 10^−5^	4 × 10^−3^	1.4 ↑	1.6 ↑	2.8 ↑	4.2 ↑	3 × 10^−6^	5 × 10^−3^
	*TNFAIP6*	206026_s_at	1.8 ↑	3 × 10^−4^	8 × 10^−3^	1.3 ↑	2.3 ↑	3 × 10^−5^	2 × 10^−3^	1.1 ↑	1.4 ↑	2.0 ↑	2.8 ↑	5 × 10^−5^	2 × 10^−3^

Legend: ID—identification number; CRC—all colorectal cancer samples against all controls; *p*—probability; BH—Benjamini–Hochberg correction; LCS—low clinical stage; HCS—high clinical stage; CSI, CSII, CSIII, CSIV—clinical stages in colorectal development; *FC*—fold change against control; Regulation: ↑ upregulation, ↓ downregulation.

**Table 3:** Mutual correlations between distinguished significant gene expression.

A						B						C					
Control vs. Control						Control vs. CRC						CRC vs. CRC					
Gene	vs.	Gene		r	*p*	Gene	vs.	Gene		r	*p*	Gene	vs.	Gene		r	*p*
*GNA15*	⇄	*HRH3*	+	0.63	0.00	*HRH2*	*⇄*	*GNA15*	*+*	0.50	0.03	*GNA15*	*⇄*	*HRH2*	*+*	0.53	0.02
*HRH3*	⇄	*CXCL3*	+	0.48	0.04	*HRH4*	*⇄*	*GNA15*	*−*	−0.58	0.01	*GNA15*	*⇄*	*MAOA*	*−*	−0.48	0.04
*HRH4*	⇄	*MAOA*	+	0.56	0.02	*HRH4*	*⇄*	*SPHK1*	*−*	−0.48	0.05	*GNA15*	*⇄*	*CXCL8*	*+*	0.68	0.00
*HRH4*	⇄	*WASF2*	−	−0.48	0.04	*HRH4*	*⇄*	*TNFAIP6*	*−*	−0.67	0.00	*GNA15*	*⇄*	*SPHK1*	*+*	0.68	0.00
*HRH4*	⇄	*AEBP1*	−	−0.63	0.01	*MAOA*	*⇄*	*TNFAIP6*	*−*	−0.58	0.01	*GNA15*	*⇄*	*TNFAIP6*	*+*	0.57	0.01
*HRH4*	⇄	*SPHK1*	−	−0.58	0.04	*WASF2*	*⇄*	*GNA15*	*+*	0.47	0.05	*HRH2*	*⇄*	*WASF2*	*−*	−0.55	0.02
*MAOA*	⇄	*WASF2*	−	−0.60	0.01	*WASF2*	*⇄*	*AEBP1*	*+*	0.56	0.02	*HRH4*	*⇄*	*SPHK1*	*−*	−0.48	0.05
*MAOA*	⇄	*AEBP1*	−	−0.71	0.00	*WASF2*	*⇄*	*SPHK1*	*+*	0.54	0.02	*MAOA*	*⇄*	*WASF2*	*+*	0.54	0.02
*MAOA*	⇄	*SPHK1*	−	−0.57	0.01	*WASF2*	*⇄*	*TNFAIP6*	*+*	0.60	0.01	*MAOA*	*⇄*	*SPHK1*	*−*	−0.61	0.01
*MAOA*	⇄	*TNFAIP6*	−	−0.51	0.03	*AEBP1*	*⇄*	*TNFAIP6*	*+*	0.55	0.02	*MAOA*	*⇄*	*TNFAIP6*	*−*	−0.48	0.04
*WASF2*	⇄	*AEBP1*	+	0.74	0.00	*CXCL1*	*⇄*	*CXCL2*	*+*	0.48	0.04	*WASF2*	*⇄*	*SPHK1*	*+*	0.54	0.02
*WASF2*	⇄	*SPHK1*	+	0.60	0.00	*CXCL1*	*⇄*	*CXCL3*	*+*	0.54	0.02	*AEBP1*	*⇄*	*SPHK1*	*+*	0.69	0.00
*AEBP1*	⇄	*SPHK1*	+	0.69	0.00	*CXCL3*	*⇄*	*CXCL2*	*+*	0.52	0.03	*AEBP1*	*⇄*	*TNFAIP6*	*+*	0.50	0.04
*AEBP1*	⇄	*TNFAIP6*	+	0.47	0.05	*CXCL3*	*⇄*	*CXCL3*	*+*	0.54	0.02	*CXCL1*	*⇄*	*CXCL2*	*+*	0.79	0.00
*CXCL1*	⇄	*CXCL2*	+	0.63	0.00	*CXCL3*	*⇄*	*AEBP1*	*−*	−0.51	0.03	*CXCL1*	*⇄*	*CXCL3*	*+*	0.85	0.00
*CXCL1*	⇄	*CXCL3*	+	0.79	0.00							*CXCL1*	*⇄*	*CXCL8*	*+*	0.62	0.00
*CXCL1*	⇄	*CXCL8*	+	0.53	0.02							*CXCL2*	*⇄*	*CXLC3*	*+*	0.88	0.00
*CXCL2*	⇄	*CXCL3*	+	0.73	0.00							*CXCL8*	*⇄*	*SPHK1*	*+*	0.65	0.00
*CXCL2*	⇄	*CXCL8*	+	0.82	0.00							*CXCL8*	*⇄*	*TNFAIP6*	*+*	0.58	0.01
*CXCL2*	⇄	*TNFAIP6*	+	0.61	0.01							*SPHK1*	*⇄*	*TNFAIP6*	*+*	0.72	0.00
*CXCL3*	⇄	*CXCL8*	+	0.50	0.03												
*CXCL8*	⇄	*SPHK1*	+	0.55	0.02												
*CXCL8*	⇄	*TNFAIP6*	+	0.78	0.00												
*SPHK1*	⇄	*TNFAIP6*	+	0.55	0.02												

Legend: CRC—colorectal cancer; vs.—versus; r—correlation coefficient; *p*—probability; + positive correlation; − negative correlation.

**Table 4:** Demographic and clinical characteristics of participants.

Parameters	CRC	Control
Age [years]	51–79	52–76
Male [%]	50	56.6 (6)
Haemoglobin [g/dL]	9.27–11.59	10.15–11.62
WBC ×10^3^/µL	7–10.5	5–10
Platelete [number/µL]	150,000–350,000	150,000–400,000
Glycemia [mg/dL]	73–115	80–110
Creatinine [mg/dL]	0.86–1.10	0.95–1.15
PT _INR_	0.8–1.2	0.85–1.15
APTT [s]	26–36	27–38
CRP [mg/dL]	2–6	1–5

Legend: WBC—white blood cells, PT—prothrombin time, APTT—activated partial thromboplastin time, CRP—C-reactive protein; results are presented as a range.

**Table 5:** Characteristics of samples.

Samples		Clinical Stage	Clinical features	Histopathological Grading (% of All)
CRC	LCS	CSI	T1N0M0	G2 (75), G1 (25)
		CSII	T3N0M0	G1 (60), G2 (40)
	HCS	CSIII	T3N1M0, T3N2M0	G1 (60), G3 (40)
		CSIV	Any T Any N M1a, Any T Any N M1b	G2 (75), G3 (25)
Control	Tissues without signs of neoplastic changes under the microscope, histopathologically normal large intestine			

Legend: CRC—all colorectal cancer samples; LCS—low clinical stage; HCS—high clinical stage; CS—clinical stage, T—tumour, N—node, M—metastasis, G—grading.

## Data Availability

The data are stored and available in plgrid.pl infrastructure which was created as part of the PL-Grid Life Science project for the Polish Scientific Community.

## Supplementary Materials

The following supporting information can be downloaded at: https://www.mdpi.com/article/10.3390/ijms24054913/s1.
