# Supplementary material for: Histaminergic System and Inflammation-Related Genes in Normal Large Intestine and Adenocarcinoma Tissues: Transcriptional Profiles and Relations
Source: Int J Mol Sci. 2023 Mar 3;24(5):4913. doi: 10.3390/ijms24054913 (PMC10002554; doi:10.3390/ijms24054913)
Supplement: Supplementary file 1 [file ijms-24-04913-s001.zip › Caption for tables and figures.pdf]

Table S1: Histaminergic system entities list used in the work.
Table S2: Inflammation-related entities list used in the work.
Table S3A–F: Data of moderated *t*-test (CRC, (A,B)) and *ANOVA* (LCS–HCS, C and D, CSI–CSIV, E and F) for histaminergic system (A,C,E) and inflammation (B,D,F)

Figure S1: Box–whisker plots of histaminergic system (133 mRNAs; A,C,E) and inflammation (627 mRNAs; B,D,F).
Figure S2: Profile plots of histaminergic system (133 mRNAs; A,C,E) and inflammation (627 mRNAs; B,D,F).
Figure S3: Self-organizing maps of histaminergic system (133 mRNAs; A,C,E) and inflammation (627 mRNAs; B,D,F).
