# Supplementary figures and images for: Histaminergic System and Inflammation-Related Genes in Normal Large Intestine and Adenocarcinoma Tissues: Transcriptional Profiles and Relations
Source: Int J Mol Sci. 2023 Mar 3;24(5):4913. doi: 10.3390/ijms24054913 (PMC10002554; doi:10.3390/ijms24054913)

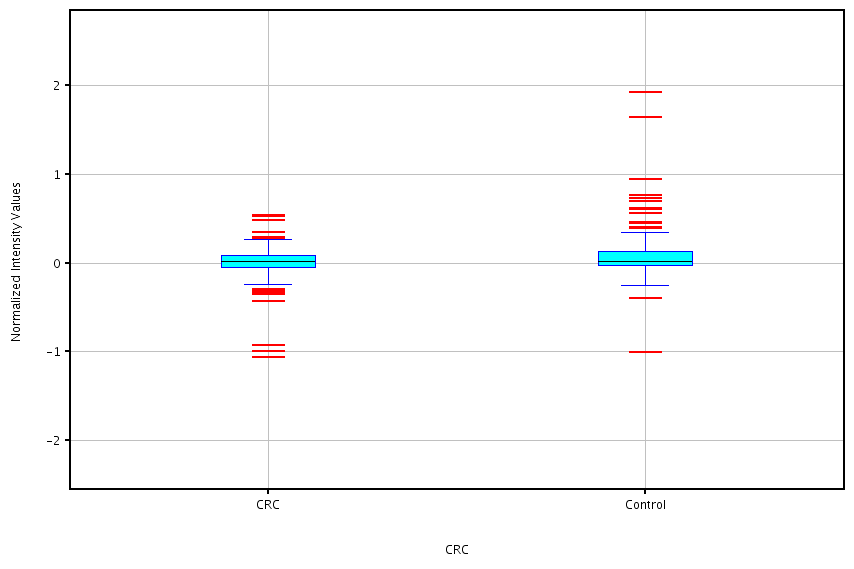

Supplement: Supplementary file 1 [file ijms-24-04913-s001.zip › Figure S1. A.png]

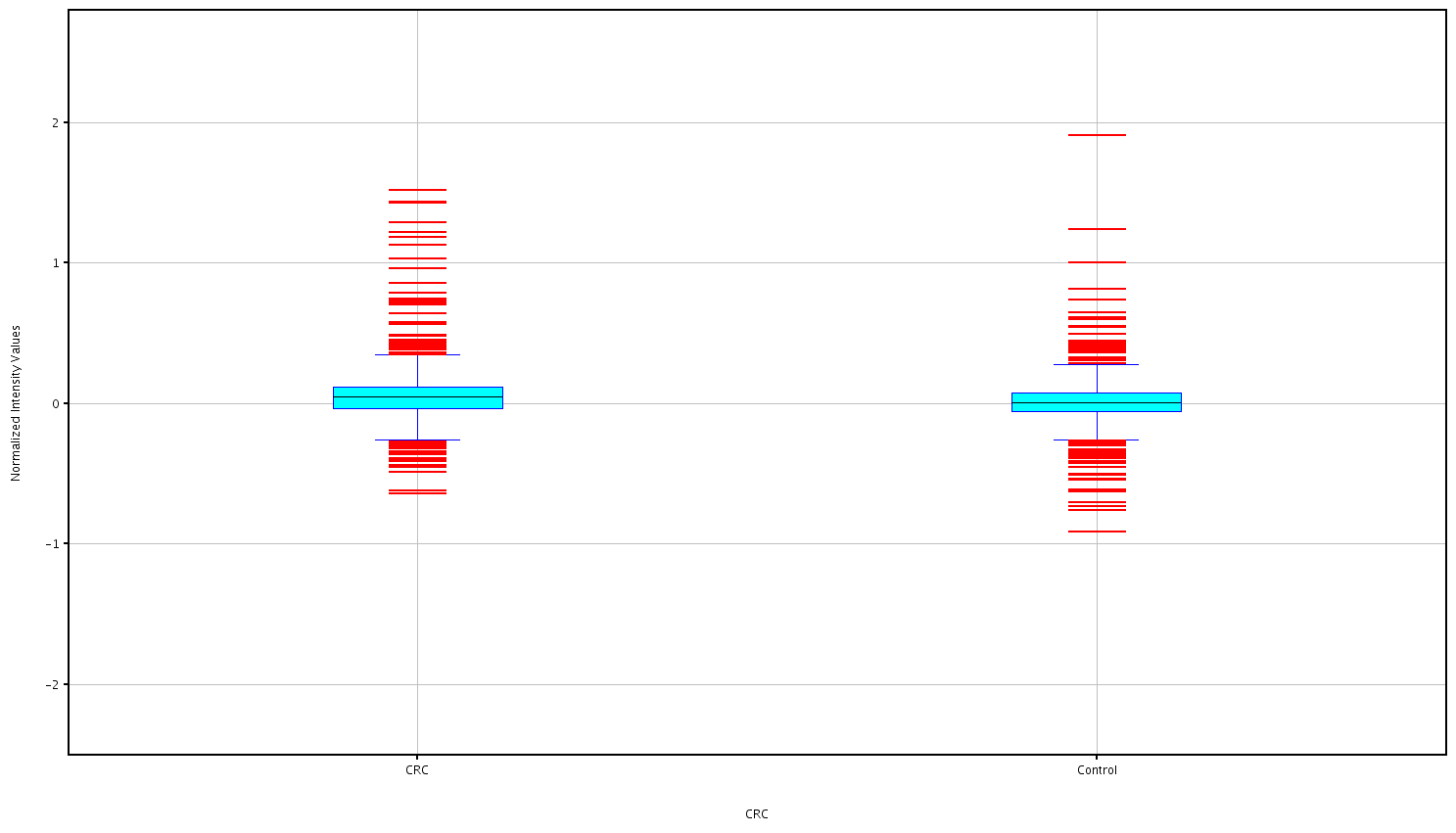

Supplement: Supplementary file 1 [file ijms-24-04913-s001.zip › Figure S1. B.png]

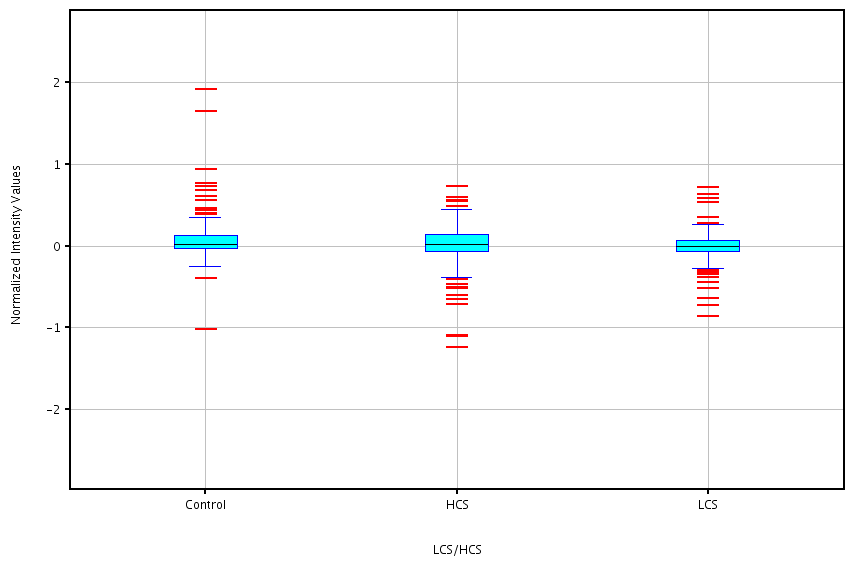

Supplement: Supplementary file 1 [file ijms-24-04913-s001.zip › Figure S1. C.png]

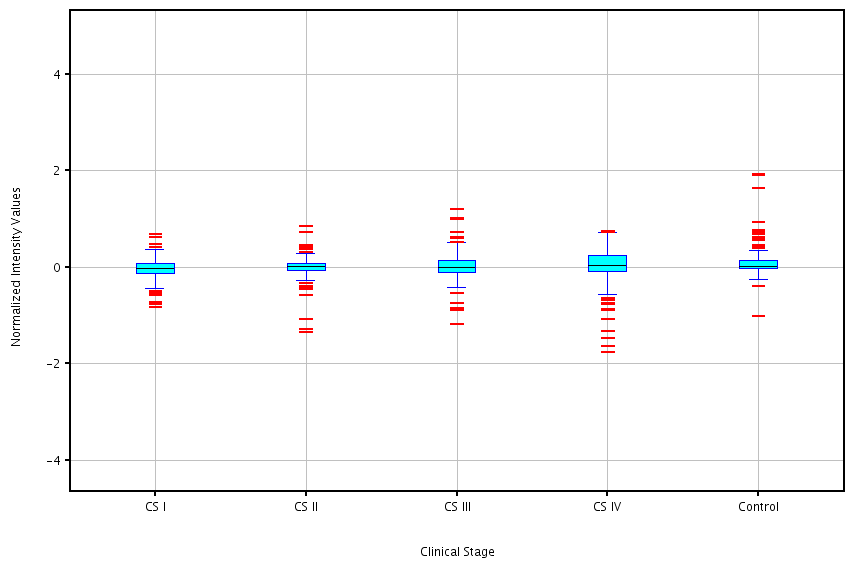

Supplement: Supplementary file 1 [file ijms-24-04913-s001.zip › Figure S1. E.png]

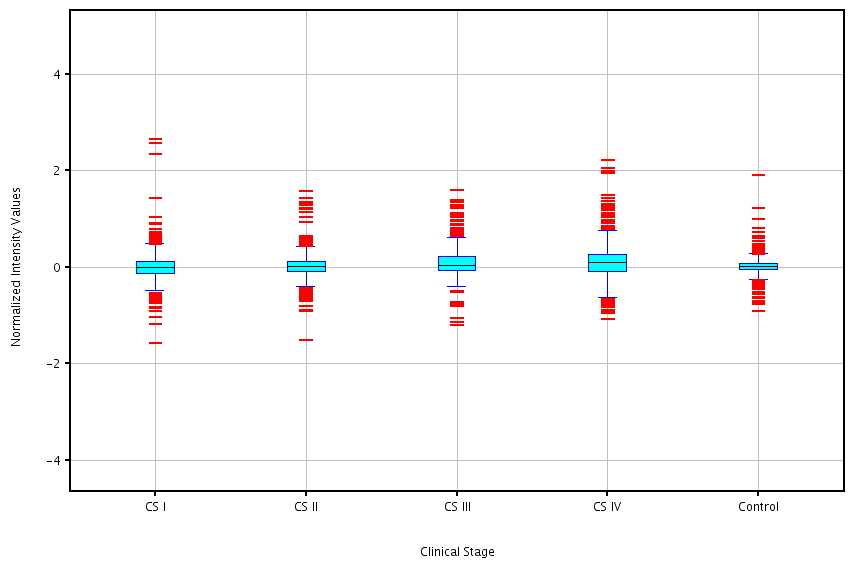

Supplement: Supplementary file 1 [file ijms-24-04913-s001.zip › Figure S1. F.png]

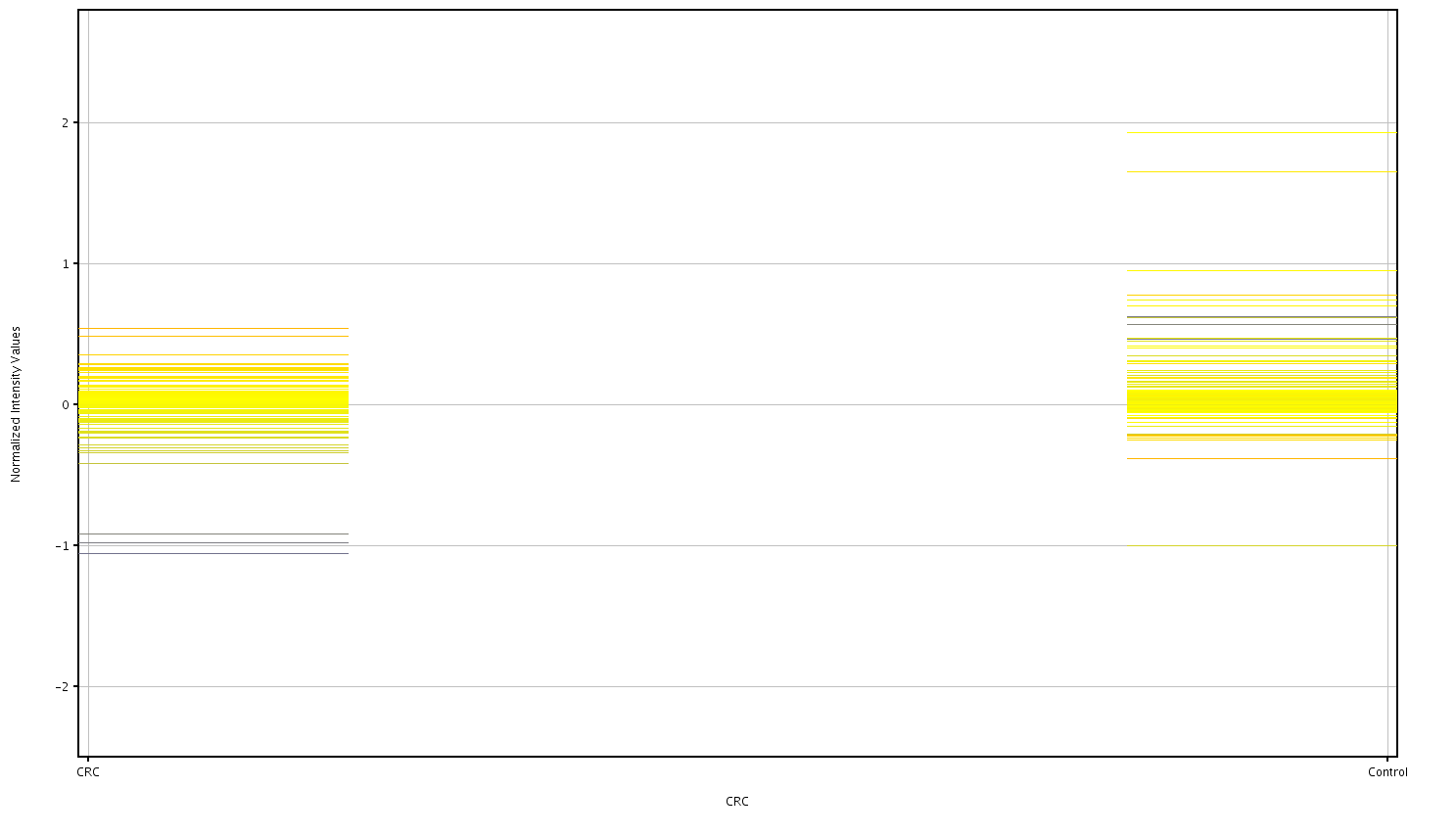

Supplement: Supplementary file 1 [file ijms-24-04913-s001.zip › Figure S2. A.png]

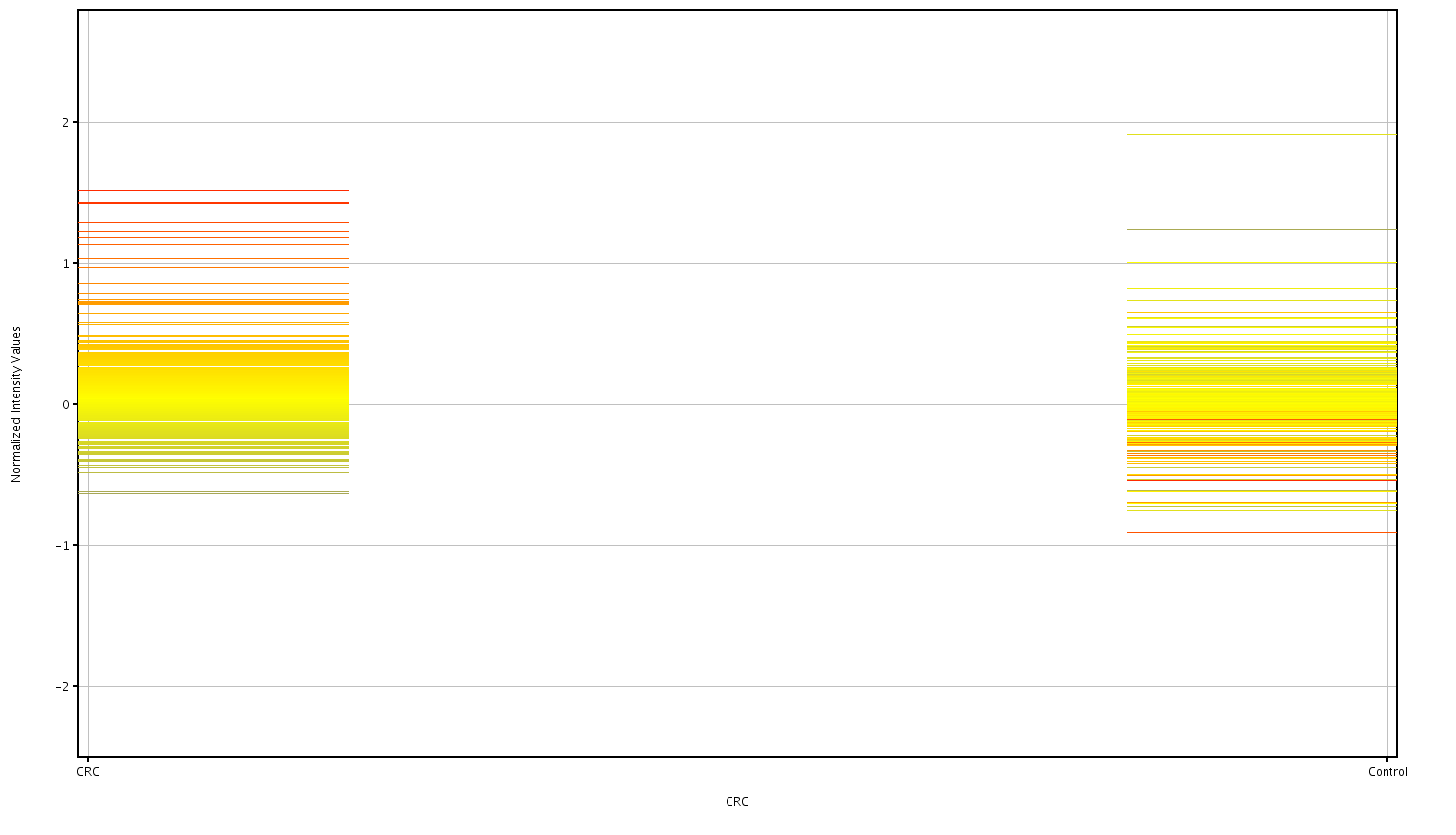

Supplement: Supplementary file 1 [file ijms-24-04913-s001.zip › Figure S2. B.png]

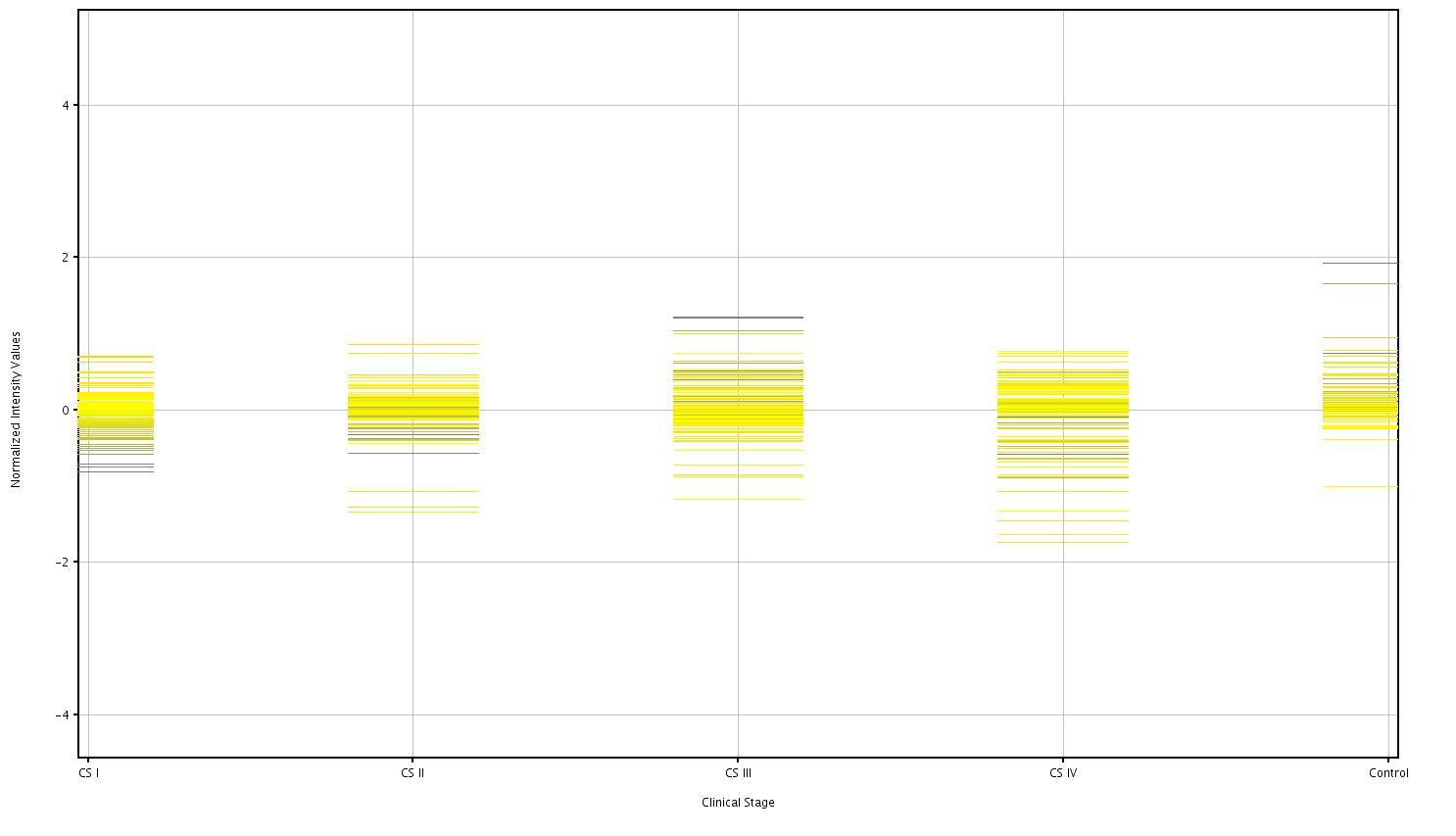

Supplement: Supplementary file 1 [file ijms-24-04913-s001.zip › Figure S2. E.jpg]

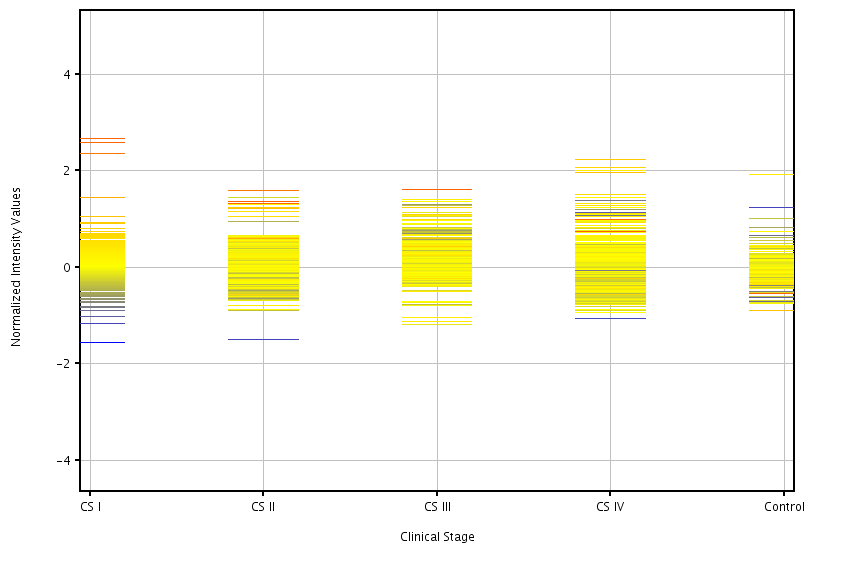

Supplement: Supplementary file 1 [file ijms-24-04913-s001.zip › Figure S2. F.png]

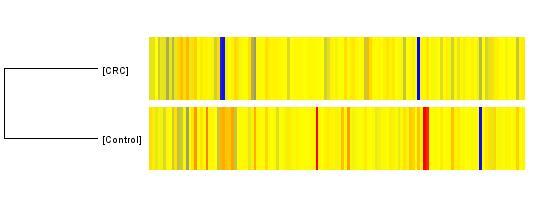

Supplement: Supplementary file 1 [file ijms-24-04913-s001.zip › Figure S3. A.jpg]

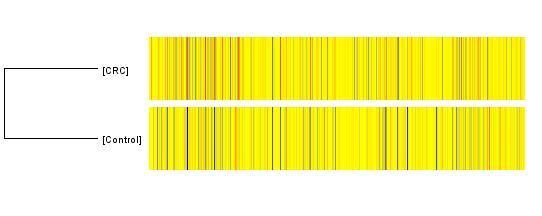

Supplement: Supplementary file 1 [file ijms-24-04913-s001.zip › Figure S3. B.jpg]

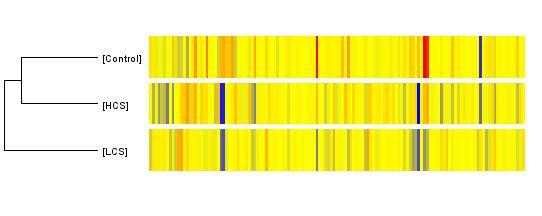

Supplement: Supplementary file 1 [file ijms-24-04913-s001.zip › Figure S3. C.jpg]

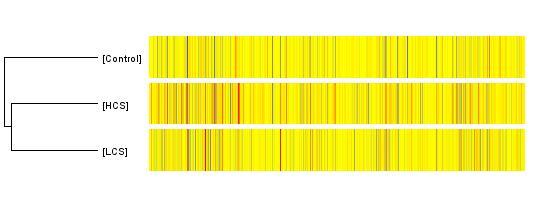

Supplement: Supplementary file 1 [file ijms-24-04913-s001.zip › Figure S3. D.jpg]

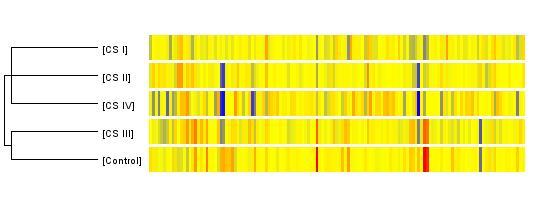

Supplement: Supplementary file 1 [file ijms-24-04913-s001.zip › Figure S3. E.jpg]

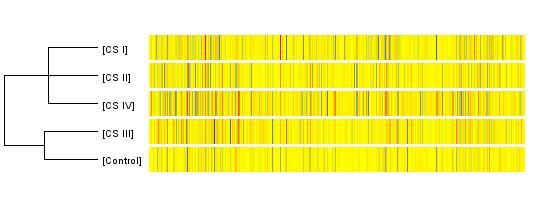

Supplement: Supplementary file 1 [file ijms-24-04913-s001.zip › Figure S3. F.jpg]
